# Neocortical Chandelier Cells Developmentally Shape Axonal Arbors through Reorganization but Establish Subcellular Synapse Specificity without Refinement

**DOI:** 10.1523/ENEURO.0057-17.2017

**Published:** 2017-05-12

**Authors:** André Steinecke, Ellie Hozhabri, Stephen Tapanes, Yugo Ishino, Hongkui Zeng, Naomi Kamasawa, Hiroki Taniguchi

**Affiliations:** 1 Development and Function of Inhibitory Neural Circuits, Max Planck Florida Institute for Neuroscience, Jupiter, FL 33458; 2 Electron Microscopy Facility, Max Planck Florida Institute for Neuroscience, Jupiter, FL 33458; 3 Allen Institute for Brain Science, Seattle, WA 98103

**Keywords:** axon initial segment, axonal remodeling, chandelier cell, cortical interneuron, subcellular synapse specificity

## Abstract

Diverse types of cortical interneurons (INs) mediate various kinds of inhibitory control mechanisms to balance and shape network activity. Distinct IN subtypes develop uniquely organized axonal arbors that innervate different subcellular compartments of excitatory principal neurons (PNs), which critically contribute to determining their output properties. However, it remains poorly understood how they establish this peculiar axonal organization and synaptic connectivity during development. Here, taking advantage of genetic labeling of IN progenitors, we examined developmental processes of axonal arbors and synaptic connections formed by murine chandelier cells (ChCs), which innervate axon initial segments (AISs) of PNs and thus powerfully regulate their spike generation. Our quantitative analysis by light microscopy revealed that ChCs overgrow and subsequently refine axonal branches as well as varicosities. Interestingly, we found that although a significant number of axonal varicosities are formed off AISs in addition to on AISs, presynaptic markers are predominantly colocalized with those on AISs throughout development. Immunoelectron microscopic (IEM) analysis also demonstrated that only varicosities apposed to AISs contain presynaptic profiles. These results suggest that subcellular synapse specificity of ChCs is genetically predetermined while axonal geometry is shaped through remodeling. Molecular cues localized at AISs may regulate target recognition and synapse formation by ChCs.

## Visual Abstract

**Figure:**
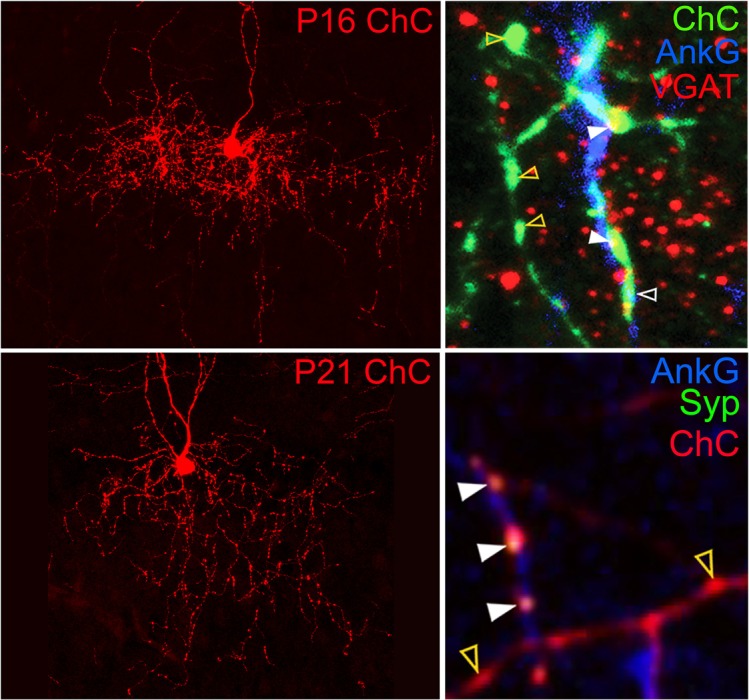

## Significance Statement

Highly structured axonal arbors with subcellular synapse specificity are one of the most striking features of cortical interneuron (IN) circuits. However, little is known about the developmental processes through which IN subtypes establish axonal and synaptic organization. Addressing this question required technical innovations that allowed us to follow the developmental time course of a uniform IN subtype at a single cell level. Using genetic targeting of chandelier cell (ChC) progenitors, we provided evidence that developing ChCs form synapses specifically on their subcellular targets, axon initial segments (AISs) of PNs, despite transient overgrowth of axonal branches and varicosities. Our results suggest that subcellular synapse specificity of ChCs is genetically predetermined while axonal geometry is established through refinement.

## Introduction

Higher-order cortical functions such as sensory processing, learning, and memory rely on precise wiring of cortical circuits that consist of excitatory principal neurons (PNs) and inhibitory interneurons (INs). Cortical INs not only balance overall network activity but also control PN spatiotemporal activity patterns, which are essential for cortical processing and representation of information (Markram et al., 2004; Klausberger and Somogyi, 2008; Kepecs and Fishell, 2014). Despite such a critical role for INs in cortical network operations, the developmental principles for IN wiring are poorly understood.

INs comprise diverse classes, which differ in morphology, physiology, gene expression, and connectivity, enabling various types of neural computations (Jonas et al., 2004; Markram et al., 2004; DeFelipe et al., 2013; Kepecs and Fishell, 2014). Mature IN subtypes display unique geometry of axonal arbors and specifically deploy synaptic terminals to distinct subcellular domains [e.g., dendrites, somata, or axon initial segments (AISs)] of PNs (Somogyi et al., 1998; Karube et al., 2004; Huang et al., 2007; Jiang et al., 2015). Such highly specialized axonal and synaptic organization contributes to determining the output properties of each IN subtype. Despite this crucial anatomic basis for inhibitory circuit function, the developmental processes of IN wiring are largely unknown. Addressing this question has been hampered by the extreme diversity of cell types and the lack of methods to target morphologically uniform IN subtypes. In addition, inability to clearly visualize subdomains of PNs has prevented our understanding of IN wiring mechanisms.

Cortical chandelier cells (ChCs) are one of the most distinct and uniform IN subtypes (Howard et al., 2005; Woodruff et al., 2010). ChC axons exhibit a characteristic geometry with many prominent vertical branches, whose terminals are specialized into strings of synaptic varicosities (cartridges) directly apposed to AISs of PNs (Jones, 1975; Szentagothai, 1975; Somogyi, 1977). Because the AIS is the site of action potential initiation, ChCs can have decisive control over spike generation in a PN ensemble, thereby regulating synchrony and oscillation of network activity (Klausberger et al., 2003; Szabadics et al., 2006; Glickfeld et al., 2009). The striking stereotypy and specificity of this axonal and synaptic organization make ChCs an ideal system to study basic cellular events of IN wiring, such as axonal branching and subcellular synapse targeting. Another advantage is that individual AISs, which can be labeled with AIS-specific markers such as anti-AnkyrinG (AnkG) antibodies, can be unambiguously identified because they are spatially separable from neighboring AISs (Jenkins and Bennett, 2001; Taniguchi et al., 2013).

Several studies established genetic strategies to reliably label ChCs, which target ChC progenitors in the medial ganglionic eminence (MGE; Inan et al., 2013; Taniguchi et al., 2013). Young ChCs migrate along stereotyped routes with a defined schedule to settle at the border between layer 1 (L1) and L2, and in L5/6 (Taniguchi et al., 2013). ChCs mostly establish mature axonal and synaptic organization by postnatal day (P)18 and modify their arbors with minor lateral axonal expansions and small increases in the number of cartridges toward P30 (Inan et al., 2013). However, the developmental processes through which ChCs establish unique axonal organization and subcellular synapse specificity have not been explored.

In this study, taking advantage of genetic labeling of ChC progenitors, we examined the developmental time course of axonal and synaptic organization and determined how axonal geometry and subcellular synapse specificity of ChCs emerge during postnatal development. We found that ChCs form excessive axonal branches and varicosities, which are remodeled via pruning. Furthermore, we demonstrate that although ChCs develop exuberant varicosities off AISs as well as on AISs, their synapses are specifically formed at AIS varicosities. Our results indicate that subcellular synapse specificity of ChCs is genetically programmed while axonal arbors are established through reorganization.

## Materials and Methods

All experimental procedures using live animals were approved by the IACUC of Max Planck Florida Institute for Neuroscience and performed in accordance with institutional and federal guidelines. The mice were kept under 12/12 h light/dark cycles and housed in standard cages with water and food ad libitum. Embryonic day 0 (E0) and P0 are defined by the day of plug and the day of birth, respectively.

### Mouse strains

*Nkx2.1-CreER* (JAX Stock 014552) and *LSL-RFP* (*Ai14*; JAX Stock 007914) mice are available from The Jackson Laboratory. In the LSL-triple green fluorescent protein (*LSL-tGFP*) mouse line, the *Rosa26* locus has been modified by targeted insertion of a construct containing the strong and ubiquitously active *CAG* promoter, a *loxP*-flanked stop cassette, a triple *GFP* gene unit, a woodchuck hepatitis virus posttranscriptional regulatory element (*WPRE*), and a *poly A* signal. The triple *GFP* unit was generated by gene synthesis (Genscript) and contains sequences for Emerald GFP (Life Technologies), TagGFP2 (Evrogen) and humanized *Renilla* GFP variants, which have been linked together by viral T2A and P2A oligopeptide sequences. The *LSL-tGFP* targeting construct was transfected into the 129S6B6F1 hybrid ES cell line G4, and correctly targeted clones were identified by PCR and Southern blot screening, then injected into C57BL/6J blastocysts to obtain chimeras for eventual germline breeding. The resulting mice were crossed to the *Rosa26-PhiC31* line (JAX Stock #007743) to delete the *PGK-Neo* selection cassette through PhiC31-mediated recombination between the *Att*B and *AttP* recombinase sites in the germline of the mice. *Ai14/+* and *LSL-tGFP/+* mouse strains were backcrossed with Swiss Webster (SW) mice for at least three generations before obtaining homozygotes. The *Ai14/Ai14* and *LSL-tGFP/LSL-tGFP* homozygous colonies were maintained by inbreeding. *Nkx2.1-CreER;Ai14/Ai14* and *Nkx2.1-CreER; LSL-tGFP/LSL-tGFP* mouse lines, which originally had had a mixed genetic background (SW and 129/B6), were outbred with the *Ai14/Ai14* and *LSL-tGFP/LSL-tGFP* homozygotes, respectively, for more than three generations. To obtain *Nkx2.1-CreER;Ai14/+* and *Nkx2.1-CreER; LSL-tGFP/+* mice for experiments, *Nkx2.1-CreER;Ai14/Ai14* and *Nkx2.1-CreER; LSL-tGFP/LSL-tGFP* male mice, respectively, were crossed with SW female mice. Both males and females were used in this study.

### Tamoxifen (Tmx) induction

Tmx was administered to timed pregnant SW females that were bred to *Nkx2.1-CreER;Ai14/Ai14 or Nkx2.1-CreER; LSL-tGFP/LSL-tGFP* males by oral gavage at E17 to induce CreER activity in the offspring. To achieve sparse labeling of ChCs the dose was adjusted to 0.15 mg/30 g of body weight. Tmx solution was prepared at a working concentration of 2 mg/ml in corn oil (Sigma), protected from light, and kept refrigerated for no longer than one month.

### Electroporation of dissociated MGE cells and transplantation

Timed pregnant SW mice were deeply anesthetized at E17 using isoflurane. After cervical dislocation the uterus was dissected and kept in AdvanceSTEM ES qualified DPBS (GE). The embryonic brains were dissected and cut into 400-µm sections using a tissue chopper (Intra Cell). Brain slices were kept in Hibernate buffer [Hibernate-E (Gibco), 100× GlutaMAX (Gibco), 50× B27 supplement (Gibco)], and the ventral MGE was dissected and collected in 1 ml Hibernate buffer. After a 10-min incubation in trypsin solution (Sigma-Aldrich) at 37°C and the addition of 10% FBS final concentration (Sigma-Aldrich) the tissue was spun down for 5 min at 500 rpm and resuspended in OptiMEM (Gibco). Subsequently, the cells were spun down as before and resuspended in 100 µl of OptiMEM containing 25 µg of *pCBH-ZsGreen* or *pCAG-DsRed + pCAG-Syp-YFP* vector DNA. The cell suspension was transferred to a cuvette (Bulldog Bio 2-mm gap aluminum cuvettes, catalog 12358-346) and electroporated (Nepa Gene; poring: 275 V, 0.3-ms duration, 50-ms interval, 5 pulses, 10% decay; transfer: 20 V, 50-ms duration, 50-ms interval, 5 pulses, 40% decay). The cells were spun down as before, resuspended in 10 µl of Hibernate buffer at a density of 100,000 cells/µl, and kept on ice.

P1 SW mice were anesthetized on ice for 5 min and the absence of pain perception was assured. A total of 10,000-20,000 cells were injected at 0.2 mm anterior and 0.2 mm lateral of bregma between 150 and 300 µm in depth using pulled glass pipets (Warner Instruments) in combination with a stereotactic apparatus (Kopf) and a picospritzer (Parker). The incision was closed with vet bond (Patterson Veterinary) and the pups were placed on a heating plate at 37°C until full recovery.

Many of the transplanted cells migrated from the somatosensory cortex (SSC) and settled in the medial prefrontal cortex (mPFC) and anterior cingulate cortex (ACC). Among transplanted ZsGreen or DsRed-expressing cells, ∼30% were ChCs; the rest were other INs (data not shown). They were largely located in expected laminar positions (L2, L5, and L6).

### Immunohistochemistry, imaging, and three-dimensional (3D) reconstruction

Mice were deeply anesthetized with an IP injection of ketamine and xylazine (50 mg/kg ketamine, Vetco, 5 mg/kg xylazine, Akorn) and transcardially perfused with 15 ml cold 0.9% saline solution followed by 20 ml 4% PFA in PBS. Brains were dissected and postfixed in 2% PFA overnight at 4°C and afterward stored in PBS until further use. 60 μm coronal brain sections were prepared using a vibratome (Leica), permeabilized in 0.5% Triton X-100 in PBS for 10 min followed by blocking in 0.1% Triton X-100, 10% donkey serum (Jackson ImmunoResearch) in PBS for 1 h. Subsequently, slices were incubated with primary antibodies in blocking solution over night at 4°C. The following primary antibodies were used: chicken anti-GFP 1:800 (Abcam, catalog ab13970), mouse anti-AnkG 1:500 (UC Davis/NIH Neuromab, catalog clone N106/36 75-146), rabbit anti-VGAT 1:3000 (Synaptic Systems catalog 131011), rabbit anti-ZsGreen 1:500 (Clonetech, catalog 632474), and rabbit anti-RFP 1:800 (Rockland, catalog 600-401-379). After three washing steps each 10 min in PBS, the slices were incubated in appropriate fluorescently labeled secondary antibodies in blocking reagent for visualization at room temperature for 2 h. All secondary antibodies were purchased from Jackson ImmunoResearch. The following secondary antibodies were used at a concentration of 0.75 μg/ml: anti-chicken IgY Alexa Fluor 488 (catalog 703-545-155), anti-mouse IgG Alexa Fluor 488 (catalog 715-545-151), anti-rabbit IgG Alexa Fluor 488 (catalog 711-545-152), anti-rabbit IgG Cy3 (catalog 711-165-152), anti-mouse IgG Cy3 (catalog 715-165-151), and anti-mouse IgG Cy5 (catalog 715-175-150). After four washing steps in PBS slices were mounted in FluoromountG mounting medium (Southern Biotech) and stored at 4°C. Low-magnification fluorescent images were captured using a microscope (Olympus BX51, 4× UPlanSApo NA: 0.16; Olympus) equipped with a CCD camera (QImaging) and QCapture acquisition software. For detailed structural analysis, z-stack images were acquired using a confocal microscope [CLSM 780 or 880 (Zeiss), 20× Plan ApoChromat, NA: 0.8 (Zeiss), 63× Plan ApoChromat oil immersion, NA: 1.4 (Zeiss)]. For local analysis of axonal arbors, images were taken from the soma-containing slice. Three-dimensional reconstruction of axons included in a 100 × 100 µm square directly below the soma was semiautomatically performed using the filament tracer tool in the Imaris software followed by manual corrections. Because L2 ChCs analyzed in this study project dendrites mostly to L1, axonal areas barely overlap with dendritic branches. Indeed, our region of interest contained no dendritic structures. The total length of the axonal trajectory and the number of branch points defined as axonal bifurcation points were measured automatically using Imaris.

For quantification of AIS and off-target varicosities, the same images as used for axonal tracing were analyzed. The Imaris spots tool was used to automatically select varicosities: after automatic image thresholding, spots with diameter of smaller than 3 µm in *x-y* and 3.5 µm in *z* were selected as varicosities. The varicosities with the highest and the lowest intensity in each image were confirmed to meet the previously used criterion for axonal varicosities (De Paola et al., 2006) that they are at least two times brighter than the adjacent axonal shafts. AnkG signals representing AISs were 3D-rendered using the Imaris surface tool: after automatic thresholding of AnkG images, objects that have bounding boxes with larger than 10 µm in height and 2 µm in width were selected. The threshold value (10 × 2 µm) was determined based on the size of a bounding box for the smallest AIS stained with AnkG antibodies. Varicosities apposed to the rendered AISs were counted as AIS varicosities. The number of off-target varicosities was obtained by subtracting the number of AIS varicosities from the total number of varicosities.

Quantification of synaptophysin (Syp)-YFP and VGAT-positive varicosities was manually performed using Fiji image analysis software. The background of Syp-YFP and VGAT images was determined by measuring the average intensity of putative cytosolic areas inside the somata. Puncta with mean gray values higher than the background level were considered Syp-YFP or VGAT signals. At least 50 varicosities per cell were analyzed for each type of varicosities (i.e., AIS and off-target varicosities) within a 100 × 100 µm square right below the soma.

### Immunoelectron microscopy (IEM)

Mice were anesthetized with ketamine xylazine mixture (50 mg/kg ketamine, 5 mg/kg xylazine) and perfused transcardially with 0.9% NaCl in 0.1 M Sorensen’s phosphate buffer (PB; pH 7.4) followed by 4% paraformaldehyde, 0.25-0.5% glutaraldehyde in 0.1 M PB for 12 min; 50-µm-thick coronal sections of the cortex were obtained on a vibratome (Leica). GFP expressing single isolated ChCs were identified using an epi-fluorescence microscope [Olympus BX51 10× UPlanSApo NA: 0.4 (Olympus) or 20× UPlanFl NA: 0.5 (Olympus)] and slices containing single GFP expressing ChCs were selected for follow-up processing. After post-fixation in 4% PFA in PB for 1h and washing in 0.1 M PB, sections were treated with 15% and 30% sucrose in 0.1 M PB. Sections were permeabilized by submersion in liquid nitrogen followed by incubation in a blocking solution of 10% normal goat serum (NGS), 1% fish skin gelatin (FSG) in 50 mM Tris buffered saline (TBS) for 1 h. Sections were incubated with a rabbit anti-GFP antibody (0.25 µg/ml, Abcam, ab#6556) diluted in TBS with 1% NGS, 0.1% FSG, washed with TBS and then incubated with a nanogold-conjugated anti-rabbit antibody (1:50, catalog 2003, Nanoprobe) diluted in TBS with 1% NGS, 0.1% FSG. Immunogold-labeled sections were washed and silver enhanced using HQ Silver intensification kit (Nanoprobe, catalog 2012), then osmificated with 0.5% OsO_4_, en bloc stained with 1% uranyl acetate, and dehydrated with a series of ethanol and acetone. Dehydrated sections were embedded in Fluka Durcupan resin (Sigma-Aldrich) and polymerized at 60°C for 2 d. Immunogold-labeled ChCs were trimmed out under a dissecting scope and serially sectioned at 45-nm thickness with an ultramicrotome (UC7, Leica). Serial ultrathin sections were counter stained with uranyl acetate and lead citrate. Samples were examined in a Tecnai G2 Spirit electron microscope (FEI) at 100 kV. Images were acquired with a Veleta CCD camera (Olympus) operated by TIA software (FEI).

### Transmission electron microscopy (TEM) data collection and analysis

GFP-immunogold-positive profiles with a diameter over 200 nm were identified and those profiles were serially imaged to cover whole varicosities from neck to neck. A typical varicosity spans 13-19 serial sections. TEM Image analysis was performed with Photoshop (Adobe) and Fiji image analysis software (NIH). For 3D reconstruction the consecutive sections spanning one varicosity were aligned and the varicosity as well as the AIS structures were manually outlined. Subsequently, a 3D reconstruction image was automatically generated using Amira software (Amira 6.1.1, FEI). Vesicles in the profiles were manually marked using Photoshop and counted. The same images were opened in Fiji and the area of profiles was demarcated with the polygon tool and measured. Immunogold particles in the profiles were thresholded and subtracted from the total area. To analyze the roundness of vesicles, all vesicles in the profiles were manually demarcated using the freehand tool and roundness was defined as 4π * (area/perimeter^2^) (Herman et al., 2014).

### Statistical analyses

Data are presented as the mean ± SEM throughout experiments. All data were collected from at least three independent experiments or animals. All statistical analyses were performed using Prism 6. Normality of the data were assessed by the Shapiro-Wilk test. Differences were tested using Student’s *t test* for two groups or one-way ANOVA for more than two groups. After ANOVA a Bonferroni *post hoc* test was performed to analyze statistical significance between groups; *p* < 0.05 was considered significant. Statistical significance was presented in figures and throughout the text in the following manner: *p < 0.05, **p < 0.01, and ***p < 0.001. A summary of each statistical analysis can be found in Table 1.

**Table 1.** Statistical table

	Data structure	Type of test	Power	Experiments
a	Normal distribution	One-way ANOVA Bonferroni *post hoc*	*p* = 0.0043	Mean number of branch points
b	Normal distribution	One-way ANOVA Bonferroni *post hoc*	*p* = 0.0003	Mean number of total varicosities
c	Normal distribution	One-way ANOVA Bonferroni *post hoc*	*p* = 0.0001	Mean number of off-target varicosities
d	Normal distribution	One-way ANOVA Bonferroni *post hoc*	*p* = 0.0001	Mean number of AIS varicosities
e	Normal distribution	Student’s *t* test, two tailed, unpaired	*p* = 0.542	Mean number of varicosities/AIS
f	Normal distribution	Student’s *t* test, two tailed, unpaired	*p* = 0.244	Mean number of cartridges
g	Normal distribution	One-way ANOVA Bonferroni *post hoc*	*p* < 0.0001	Mean % of off-target varicosities
h	Normal distribution	Student’s *t* test, two tailed, unpaired	*p* < 0.0001	Mean % of VGAT+ varicosities P16
i	Normal distribution	Student’s *t* test, two tailed, unpaired	*p* = 0.0191	Mean number of branch points (transpl.)
j	Normal distribution	Student’s *t* test, two tailed, unpaired	*p* = 0.0238	Mean number of total varicosities (transpl.)
k	Normal distribution	Student’s *t* test, two tailed, unpaired	*p* < 0.0001	Mean % of off-target varicosities (transpl.)
l	Normal distribution	Student’s *t* test, two tailed, unpaired	*p* < 0.0001	Mean % of Syp+ varicosities
m	Normal distribution	One-way ANOVA Bonferroni *post hoc*	*p* = 0.0002	Mean density of vesicles P16
n	Normal distribution	One-way ANOVA Bonferroni *post hoc*	*p* < 0.0001	Mean roundness of vesicles P16
o	Normal distribution	Student’s *t* test, two tailed, unpaired	*p* < 0.0001	Mean % of VGAT+ varicosities P28
p	Normal distribution	Student’s *t* test, two tailed, unpaired	*p* = 0.78	Mean density of vesicles P28
q	Normal distribution	Student’s *t* test, two tailed, unpaired	*p* < 0.0001	Mean roundness of vesicles P28

## Results

### Axonal branches and varicosities are concurrently increased and pruned in developing ChCs

**Figure 1.**
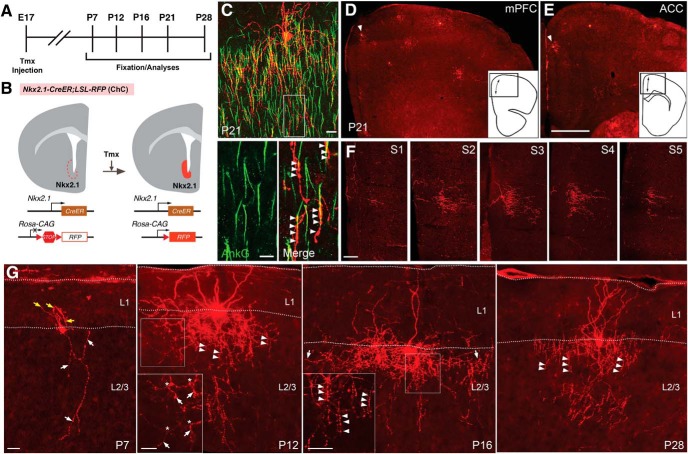
ChC axonal branches and varicosities undergo developmental refinement. ***A***, Experimental timeline. Timed pregnant females were administered with Tmx at E17 to induce Cre activity in *Nkx2.1-CreER;LSL-RFP* embryos. Pups were analyzed at each indicated postnatal time point. ***B***, Upper schematics show coronal sections in a late embryonic *Nkx2.1-CreER;LSL-RFP* brain depicting induction of RFP in the *Nkx2.1*-expressing MGE. Lower schematics denote Tmx-induced excision of a STOP cassette resulting in constitutive RFP expression. ***C***, top panel, A confocal projection image of a P21 L2 ChC (red) and AISs labeled by AnkG staining (green). Bottom panels, High-magnification confocal images of a single optical section in a boxed area in top panel. Note putative cartridges (arrowheads) associated with AISs. Scale bars, 20 μm (top) and 10 μm (bottom). ***D***, ***E***, Epifluorescent images of partial coronal sections containing the mPFC (***D***) and the ACC (***E***) of a P21 brain induced by low-dose Tmx at E17. Note that RFP+ cells (arrowheads) are sparsely labeled. Schematics in insets show whole coronal sections at the level of the mPFC and the ACC in ***D***, ***E***, respectively. The double-headed arrows show areas of the mPFC and the ACC in ***D***, ***E***, respectively. Scale bar, 200 μm. ***F***, Confocal projection images of five serial 60-μm sections spanning a whole single L2 ChC in a P21 brain induced at E17. Note that there is no overlap with other cells. Scale bar, 100 μm. ***G***, Confocal projection images of single ChCs at P7, P12, P16, and P28. White and yellow arrows in P7 show axons and presumptive immature dendrites extending downwards and upwards, respectively. Insets in P12 and P16 show confocal images of single optical sections in boxed areas. Arrowheads in the P12, P16, and P21 image indicate putative cartridges. Asterisks in a P12 ChC image denote branch points at which horizontal axons form vertical branches downward (arrows). Arrows in a P16 ChC image show axonal collaterals. Scale bars, 25 and 20 μm (insets).

To characterize the developmental processes of ChC axonal branching and synapse organization, we labeled cortical ChCs by combining *Nkx2.1-CreER* knockin mice with *loxP-stop-loxP-RFP* (*LSL-RFP*) reporter mice (Fig. 1*B*; Madisen et al., 2010; Taniguchi et al., 2011; Taniguchi et al., 2013). RFP induction in MGE progenitors at E17 predominantly labels cortical ChCs in postnatal *Nkx2.1-CreER;LSL-RFP* mice (Fig. 1*A–C*; Madisen et al., 2010; Taniguchi et al., 2013). Since labeled ChCs were most frequently found in the upper sublayer of L2 facing L1 in the mPFC and ACC, we focused on L2 ChCs in these cortical areas. To visualize axonal structures at high resolution, we optimized the dose of Tmx to a point where we could reliably label ChCs that were clearly separated from each other (Fig. 1*C–F*). Our analysis focused on branch points and varicosities, which have been used as metrics for branching and synaptogenesis, respectively.

By P7, ChCs developed one or two long and less branched vertical processes projecting toward deeper layers; they also formed several short processes from the soma, extending into L1 (Fig. 1*G*, P7, yellow arrows). By P12, ChCs began to exhibit intertwined and curved axonal processes (Fig. 1*G*, P12). Several horizontal axonal collaterals bilaterally emanated from a primary axon, forming multidirectional, dense, and complex branches into deeper layers (Fig. 1*G*, P12, arrows). At this stage, they developed only a limited number of prominent putative cartridges (Fig. 1*G*, P12, arrowheads). By P16, the size and complexity of ChC axonal arbors dramatically increased, and several axonal collaterals frequently extended horizontally (Fig. 1*G*, P16, arrows). Many putative cartridges (Fig. 1*G*, P16, arrowheads) emerged but were difficult to isolate because of numerous varicosities between them. After P21, ChCs displayed a mature axonal organization, which was characterized by regularly spaced vertical branches with prominent putative cartridges (Fig. 1*G*, P28, arrowheads). The exuberant varicosities between putative cartridges found at P16 appeared to be pruned by this stage.

**Figure 2.**
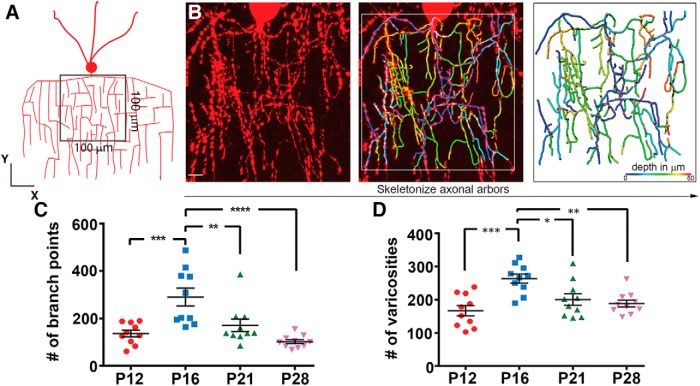
Quantitative analyses of branch points and varicosities during postnatal development. ***A***, ***B***, A strategy for quantification of ChC axonal arbors. A schematic showing a square area (100 × 100 μm) selected for axonal arbor analysis in a 60-μm-thick section containing a ChC. The square was placed directly below a soma. Three-dimensional images were reconstructed from z-stacked confocal images within the square, axonal trajectories were semiautomatically traced using Imaris with manual corrections, and axonal branches are color-coded according to their depth (***B***). See also Movie 1. ***C***, The average number of branch points in ChCs at different postnatal ages. The number of branch points at P16 is significantly greater than those at P12, P21, and P28 (*n* = 10 ChCs for each; ≥3 brains per condition; one-way ANOVA: **p < 0.01, ***p < 0.001, ****p < 0.0001)^a^. Data are presented as mean ± SEM. ***D***, The average total number of ChC axonal varicosities. The total number of varicosities at P16 is significantly greater than those at P12, P21, and P28 (*n* = 10 ChCs for each; ≥3 brains per condition; one-way ANOVA: *p < 0.05, **p < 0.01, ***p < 0.001)^b^. Data are presented as mean ± SEM.

To validate these qualitative observations, we conducted quantitative analyses of axonal arbors at P12, P16, P21, and P28 with the aid of the image analysis software Imaris. Three-dimensional images of proximal axonal arbors in a 100 × 100 μm square located immediately below the soma were reconstructed from z-stacked confocal images and skeletonized for measurement (Fig. 2*A*,*B*; Movie 1). We counted the number of branch points and varicosities. We found that both values significantly increased between P12 and P16 and decreased between P16 and P28 (Fig. 2*C*,*D*; mean number of branch points^a^: P12, 137 ± 14; P16, 290 ± 37; P21, 171 ± 26; P28, 102 ± 8; mean number of varicosities^b^: P12, 167 ± 16; P16, 263 ± 14; P21, 200 ± 17; P28, 188 ± 10; *n* = 10 ChCs for each time point; one-way ANOVA: *p < 0.05, **p < 0.01, ***p < 0.001, ****p < 0.0001). These results identify a developmental time course of axonal arborization followed by pruning in developing ChCs. They also suggest that branches and varicosities of ChCs are concurrently augmented and remodeled during the second and third postnatal weeks, respectively.

Movie 1. Axonal tracing of cortical ChCs. Three-dimensional reconstruction of 60-µm confocal z-stack of a ChC whose axonal arbor in a 100 × 100 µm square below the soma was traced using Imaris software. 10.1523/ENEURO.0057-17.2017.video.1

### Developing ChCs form AIS and off-target varicosities that undergo distinct developmental regulation

**Figure 3.**
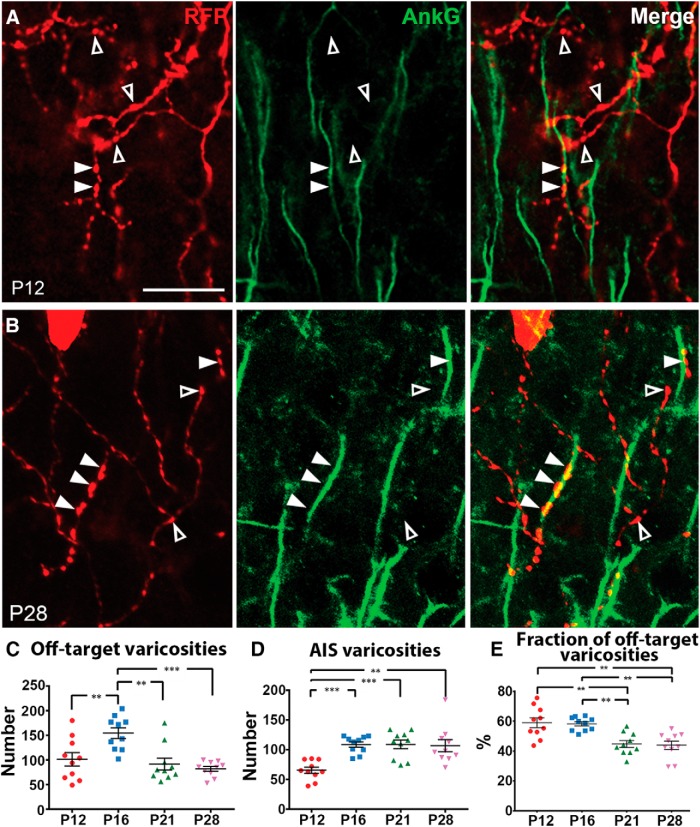
ChCs form AIS and off-target varicosities that undergo distinct developmental regulation. ***A***, ***B***, Confocal images of single optical sections showing varicosities (red) and AISs labeled by AnkG staining (green) in P12 (***A***) and P28 (***B***) brains. Filled and empty arrowheads represent AIS and off-target varicosities, respectively. Scale bar, 20 μm. See also the Movie 1. ***C***, The average number of off-target varicosities. The number of off-target varicosities at P16 is significantly greater than those at P12, P21, and P28 (*n* = 10 ChCs for each; ≥3 brains per condition; one-way ANOVA: **p < 0.01, ***p < 0.001)^c^. Data are presented as mean ± SEM. ***D***, The average number of AIS varicosities. The number of AIS varicosities at P12 is significantly lower than those at P16, P21, and P28 (*n* = 10 ChCs for each; ≥3 brains per condition; one-way ANOVA: **p < 0.01, ***p < 0.001)^d^. Data are presented as mean ± SEM. ***E***, The average percentage of off-target varicosities. The percentage of off-target varicosities at P21 and at P28 is significantly greater than those at P12 and P16 (*n* = 10 ChCs for each; ≥3 brains per condition; one-way ANOVA: **p < 0.01)^g^. Data are presented as mean ± SEM.

The numerous varicosities formed by younger ChCs (e.g., P12 and P16) make putative cartridges difficult to isolate (Fig. 1*G*, P12 and P16). This observation led to our hypothesis that young ChCs form varicosities that are not associated with AISs. To test this hypothesis, we labeled AISs with anti-AnkG antibodies, which unambiguously label AISs and enable the identification of cartridges in ChCs (Fig. 1*C*). We confirmed the reliability of our immunohistochemistry-based detection of AISs by staining brain sections containing PNs electroporated with GFP; we detected AISs of all the GFP-expressing PNs (data not shown). Interestingly, our analysis revealed that P12 ChCs formed many off-target varicosities in addition to AIS varicosities (Fig. 3*A*); in fact, off-target varicosities outnumbered AIS varicosities at P12 (Fig. 3*E*).

To characterize the developmental dynamics of off-target and AIS varicosities, we examined their average number along proximal axonal arbors at different time points (Fig. 1*A*). Off-target and AIS varicosities concurrently increased between P12 and P16 (Fig. 3*C*,*D*; mean number of off-target varicosities^c^: P12, 101 ± 14; P16, 155 ± 11; mean number of AIS varicosities^d^: P12, 66 ± 5; P16, 109 ± 5; *n* = 10 ChCs for each time point; one-way ANOVA: **p < 0.01, ***p < 0.001). However, off-target varicosities markedly decreased by half between P16 and P21 (Fig. 3*C*; mean number of off-target varicosities^c^: P16, 155 ± 11; P21, 92 ± 12; *n* = 10 ChCs for each time point; one-way ANOVA: **p < 0.01), whereas the average number of AIS varicosities remained at the same level from P16 onward (Fig. 3*D*; mean number of AIS varicosities^d^: P16, 109 ± 5; P21, 109 ± 7; P28, 107 ± 10; *n* = 10 ChCs for each time point; one-way ANOVA: *p* > 0.05). The average number of varicosities per AIS and the average number of cartridges did not change between P16 and P21 (mean number of varicosities per AIS^e^: P16, 4.9 ± 0.1; P21, 5.2 ± 0.1; Student’s *t* test: *p* = 0.542; mean number of cartridges^f^: P16, 29 ± 2; P21, 27 ± 2; Student’s *t* test: *p* = 0.244; *n* = 10 ChCs for each time point; P16: 292 cartridges, 1438 varicosities; P21: 271 cartridges, 1407 varicosities), suggesting that the formation of AIS varicosities is complete as early as P16 at least in proximal ChC axons.

Overall, these results define the developmental time frame during which ChC axons establish varicosities that are associated with AISs and exhibit the vertically elongated appearance of cartridges. They further demonstrate that developmental remodeling of varicosities is due to specific pruning of off-target varicosities that are excessively generated during axonal elaboration. It is of note that off-target varicosities occupy a significant fraction of total varicosities at least in young adult animals (e.g., P21 and P28; Fig. 3*B*,*C*,*E*; mean percentage of off-target varicosities^g^: P12, 59 ± 3%; P16, 58 ± 1%; P21, 45 ± 2%; P28, 44 ± 3%; *n* = 10 ChCs for each time point; one-way ANOVA: **p < 0.01).

### Developing ChCs form synapses at AIS but not off-target varicosities

Although axonal varicosities have been appreciated as structural indicators for presynaptic terminals (Chattopadhyaya et al., 2004; Ruthazer et al., 2006; Chattopadhyaya et al., 2007; Wu et al., 2012), nonsynaptic varicosities have also been found in transverse branches of climbing fibers in the adult cerebellum (Nishiyama et al., 2007).

**Figure 4.**
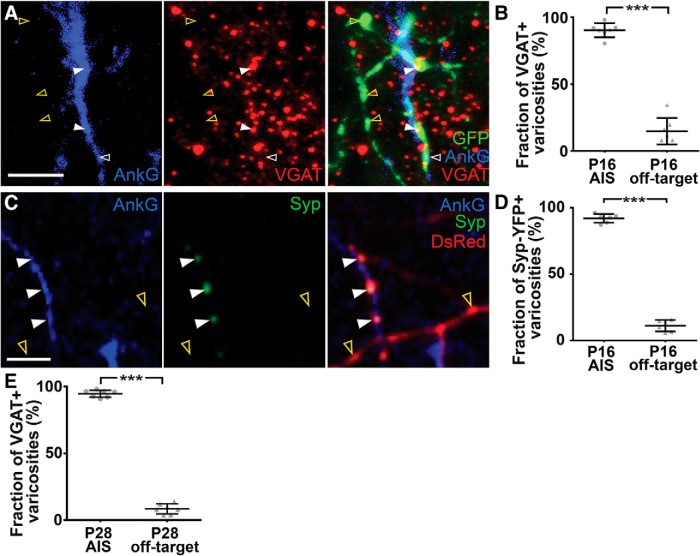
AIS but not off-target varicosities predominantly contain presynaptic markers. ***A***, Immunohistochemical analysis of VGAT localization in ChC varicosities in *Nkx2.1-CreER;LSL-tGFP* mice. Confocal images of a single optical section showing varicosities (green), VGAT (red), and AISs visualized by AnkG immunostaining (blue) in a P16 brain. White and yellow arrowheads indicate AIS and off-target varicosities, respectively. Filled and empty arrowheads show VGAT+ and VGAT- varicosities in ChC axons, respectively. Scale bar, 5 μm. ***B***, The average percentage of VGAT-containing AIS and off-target varicosities in P16 ChCs. The percentage of VGAT-containing AIS varicosities is significantly higher than that of VGAT-containing off-target varicosities (*n* = 7 ChCs for each; ≥3 brains per condition; Student’s *t* test: **p < 0.001)^h^. Data are presented as mean ± SEM. ***C***, Localization of Syp-YFP puncta in varicosities of transplanted ChCs. Confocal images of a single optical section showing AIS visualized by AnkG immunostaining (blue), Syp-YFP (green), and varicosities along the axon (red) in an EP16 brain. White and yellow arrowheads indicate AIS and off-target varicosities, respectively. Filled and empty arrowheads show Syp-YFP+ and Syp-YFP- varicosities in ChC axons, respectively. Scale bar, 5 μm. ***D***, The average percentage of Syp-YFP-containing AIS and off-target varicosities in EP16 ChCs. The percentage of Syp-YFP containing AIS varicosities is significantly higher than that of Syp-YFP containing off-target varicosities (*n* = 6 ChCs; ≥3 brains; Student’s *t* test: ***p < 0.001)^l^. Data are presented as mean ± SEM. ***E***, The average percentage of VGAT-containing AIS and off-target varicosities in P28 ChCs. The percentage of VGAT-containing AIS varicosities is significantly higher than that of VGAT-containing off-target varicosities (*n* = 7 ChCs for each; ≥3 brains per condition; Student’s *t* test: ***p < 0.001)°. Data are presented as mean ± SEM. See Extended Data Figure 4-1.

To determine whether synapses are formed exclusively at AIS varicosities or ubiquitously at both AIS and off-target varicosities during the wiring of ChC axons, we first conducted immunohistochemistry with antibodies against vesicular GABA transporter (VGAT), a marker for GABAergic synaptic vesicles, and AnkG in P16 *Nkx2.1-CreER;LSL-tGFP* mice (Fig. 4*A*). We found that a significantly higher percentage of AIS varicosities contain VGAT compared with off-target varicosities (Fig. 4*A*,*B*; mean percentage of VGAT-positive varicosities at P16^h^: AIS, 90 ± 2%; off-target, 15 ± 4%; *n* = 7 ChCs, 357 AIS varicosities, 356 off-target varicosities; Student’s *t* test: ***p < 0.001), supporting the view that developing ChCs predominantly form synapses at AIS varicosities.

10.1523/ENEURO.0057-17.2017.f4-1 Figure 4-1 Axonal branches and varicosities in transplanted ChCs undergo similar remodeling to endogenous ChCs. ***A***, Experimental timeline. E17 MGE tissues were dissected from embryonic brains and dissociated into individual cells. The MGE cells were electroporated with *pCBH-ZsGreen* and then transplanted into P1 host pups. Brain slices were prepared from brains of pups at P19 (EP16) and P24 (EP21) and stained against ZsGreen and AnkG (data not shown). ***B***, ***C***, Confocal projection images of single transplanted ChCs at EP16 (***B***) and EP21 (***C***). Arrowheads show putative cartridges. Scale bar, 20 μm. ***D***, The average number of branch points at EP16 and EP21. The number of branch points significantly decreases between EP16 and EP21 (n = 10 ChCs for each; Student’s *t* test; *p < 0.05)^i^. ***E***, The average number of total varicosities at EP16 and EP21. The number of total varicosities decreases between EP16 and EP21 (n = 10 ChCs for each; Student’s *t* test: *p < 0.05)^j^. ***F***, The average percentage of off-target axonal varicosities at EP16 and EP21. The percentage of off-target varicosities at EP21 was significantly smaller than those at EP16 (n = 10 ChCs for each; Student’s *t* test; ***p < 0.001)^k^. All data are presented as mean ± SEM.. Download Figure 4-1, TIF file.

To further validate the predominant localization of synaptic markers in AIS varicosities at high resolution, we cotransfected *ex vivo* ChC progenitors with Syp-YFP and DsRed and transplanted them into the SSC of P1 host animals (Figs. 4*C*, Fig. 4-1*A*). This approach allowed us to express genes of interest in ChCs more reliably and efficiently than *in utero* electroporation, with which it is hard to target ChC progenitors localized in the small, ventralmost domain of the subpallial ventricular/subventricular zone at late embryonic stages (e.g., E16 and E17). We confirmed that donor ChCs developed roughly 3 d later than host ChCs. For example, transplanted ChCs in P19 host mice were equivalent to endogenous P16 (EP16) ChCs (Fig. 4-1). They underwent normal developmental processes with a time course similar to endogenous ChCs (Fig. 4-1). In EP16 ChCs, a significantly higher fraction of AIS varicosities contained Syp-YFP compared with off-target varicosities (Fig. 4*C*,*D*; mean percentage of Syp-YFP-positive varicosities at EP16^l^: AIS-varicosities, 92 ± 1%; off-target varicosities, 12 ± 2%; *n* = 6 ChCs, 464 AIS varicosities, 446 off-target varicosities; Student’s *t* test: ***p < 0.001), validating the idea that developing ChCs specifically form synapses at AIS but not off-target varicosities.

**Figure 5.**
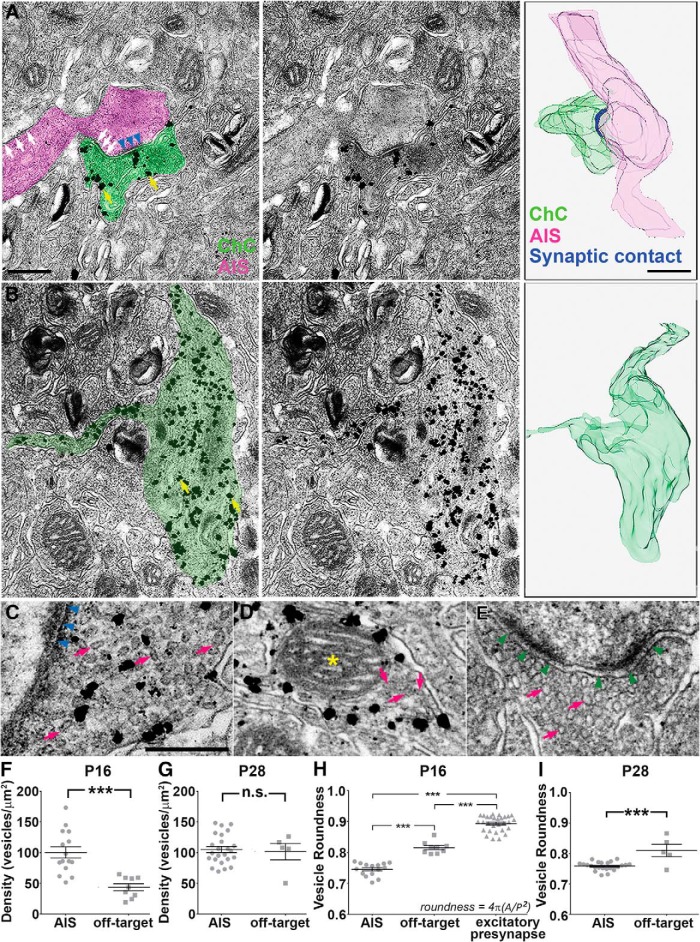
Ultrastructural characterization of AIS and off-target varicosities. ***A***, ***B***, Immunoelectron micrographs (left and middle panels) and 3D schematics reconstructed from serial EM sections (right panels) showing an AIS varicosity (***A***) and an off-target varicosity (***B***) in P16 ChCs. ChCs are tagged with tGFP in *Nkx2.1-CreER;LSL-tGFP* mice. Immunohistochemistry with anti-GFP antibodies and secondary antibodies conjugated with gold particles (yellow arrows) marks ChC axonal terminals. In left panels, varicosities and AISs are overlaid with light green and magenta, respectively. Middle panels show intact images. White arrows denote electron dense axonal membrane profiles, which are typically observed in AISs. Blue arrowheads indicate a symmetric synapse, which represents a GABAergic inhibitory synapse. Scale bar, 500 nm. Note that a clear synaptic profile is visible in an AIS varicosity, whereas there is no sign of a synapse in an off-target varicosity. ***C***–***E***, Electron micrographs showing vesicles (arrows) in an AIS varicosity (***C***), an off-target varicosity (***D***), and an excitatory presynapse (***E***) at P16. Blue and green arrowheads indicate a symmetric synapse (***C***) and an asymmetric synapse (***E***), respectively. Note distinct vesicle morphology in different types of varicosities (magenta arrows). Mitochondria (yellow asterisk in ***D***) were frequently found in varicosities. Scale bar, 250 nm. ***F***, The average density of vesicles in AIS and off-target varicosities in P16 ChCs. AIS varicosities show a higher density of vesicles than off-target varicosities (AIS, *n* = 15 varicosities; off-target, *n* = 9 varicosities; 2 ChCs per condition; Student’s *t* test: ***p < 0.001)^m^. Data are presented as mean ± SEM. ***G***, The average density of vesicles in AIS and off-target varicosities in P28 ChCs. AIS and off-target varicosities show no difference in vesicle density (AIS, *n* = 24 varicosities; off-target, *n* = 5 varicosities; 2 ChCs per condition; Student’s *t* test: n.s. *p* = 0.78)^p^. Data are presented as mean ± SEM. ***H***, The average roundness of vesicles in AIS varicosities, off-target varicosities, and excitatory presynapses at P16. AIS varicosities show the lowest roundness of vesicles, followed by off-target varicosities, and excitatory presynapses (AIS, *n* = 15 varicosities; off-target, *n* = 9 varicosities; excitatory presynapses, *n* = 29 varicosities; 2 ChCs per condition; one-way ANOVA: ***p < 0.001)^n^. Data are presented as mean ± SEM. ***I***, The average roundness of vesicles in AIS and off-target varicosities at P28. AIS varicosities show a lower roundness of vesicles than off-target varicosities (AIS, *n* = 24 varicosities; off-target, *n* = 5 varicosities; 2 ChCs per condition; Student’s *t* test: ***p < 0.001)^q^. Data are presented as mean ± SEM.

To further characterize off-target and AIS varicosities at an ultrastructural level, we performed serial section IEM analyses in *Nkx2.1-CreER;LSL-tGFP* mice at P16. We defined varicosities as axonal swellings >200 nm in diameter. To precisely characterize ultrastructural features of individual varicosities, we obtained 3D profiles through reconstruction of successive ultrathin sections (Fig. 5*A*,*B*, right panels). We identified AISs by their electron-dense membranous structures of the axon (Fig. 5*A*, white arrows; Palay et al., 1968) and considered varicosities as synaptic when they showed a parallel apposition of symmetric electron density with accumulation of synaptic vesicles (Fig. 5*A*,*C*, blue arrowheads).

As expected, we found two types of varicosities: AIS and off-target. The majority of AIS varicosities possessed clear symmetric synapses on AISs with synaptic vesicles that were of a typical GABAergic jellybean shape (Angaut and Sotelo, 1989; Fig. 5*A*,*C*; percentage of synaptic varicosities: AIS, 71%, *n* = 21 varicosities). In contrast, off-target varicosities were not associated with the AIS (Fig. 5*B*,*D*), and the structures with which they were associated were neuropil, not AISs and somata. Unlike AIS synaptic varicosities, off-target varicosities showed no sign of synapses but contained many round vesicles (Fig. 5*B*,*D*; percentage of synaptic varicosities: off-target, 0%, *n* = 9 varicosities). Notably, more than half of varicosities along ChC axons contained mitochondria (Fig. 5*D*, yellow asterisk; percentage of varicosities containing mitochondria: AIS synaptic varicosities, 66.7%, *n* = 15 varicosities; off-target varicosities, 55.6%, *n* = 9 varicosities), regardless of their type, suggesting that they are not artificial structures generated during sample preparation.

To rule out the possibility that off-target, nonsynaptic varicosities were observed because of uneven detection of AISs and/or synapses, we performed quantitative morphologic analyses of vesicles in varicosities. Specifically, we quantified vesicle density and roundness in AIS and off-target varicosities. AIS-synaptic varicosities showed significantly higher density of vesicles compared with off-target varicosities at P16 (Fig. 5*C*,*D*,*F*; mean density of synaptic vesicles at P16^m^: AIS, 101 ± 9/µm^2^, *n* = 15 varicosities; off-target, 44 ± 6/µm^2^, *n* = 9 varicosities; Student’s *t* test: ***p < 0.001). To analyze roundness, we included vesicles in excitatory presynaptic terminals as a control because they represent a round morphology (Fig. 5*E*, red arrows), which is in contrast to jellybean-like GABAergic vesicles. Our quantification indicated that excitatory vesicles have the highest roundness, followed by off-target and AIS varicosities (Fig. 5*C–E*,*H*; mean roundness at P16^n^: excitatory presynapse, 0.893 ± 0.001, *n* = 29 presynaptic terminals, 117 vesicles; off-target, 0.82 ± 0.01, *n* = 9 varicosities, 157 vesicles; AIS, 0.75 ± 0.01, *n* = 15 varicosities, 375 vesicles; one-way ANOVA: ***p < 0.001). These quantitative morphologic data suggest that AIS and off-target varicosities have distinct ultrastructural features, negating the possibility that they are artificially classified due to ambiguous identifications of AISs and synaptic specializations on IEM. Taken together, these results suggest that although developing ChCs form AIS and off-target varicosities, synapses are specifically formed at AIS varicosities.

### ChCs maintain synapse specificity in young adulthood

As described above, a significant number of off-target varicosities remain into young adulthood (e.g., P21 and P28). One could argue that they mature into synapses by early adult stages. To test this possibility, we examined synaptic properties of axonal varicosities at P28 by immunohistochemistry with VGAT antibodies and IEM analyses.

Similarly to P16 ChCs, <10% of off-target varicosities exhibited VGAT signals while 94% of AIS varicosities did so (Fig. 4*E*; mean percentage of VGAT-positive varicosities at P28^°^: AIS, 95 ± 1%; off-target, 9 ± 1%; *n* = 7 ChCs, 398 AIS varicosities, 353 off-target varicosities; Student’s *t* test: ***p < 0.001). Consistent with this result from a light microscopic analysis, our IEM analyses demonstrated that AIS but not off-target varicosities possess synaptic features defined by accumulation of synaptic vesicles near plasma membranes and a cleft flanked with parallel electron dense membranes (percentage of synaptic varicosities at P28: AIS 100%, *n* = 24 varicosities; off-target, 0%, *n* = 5 varicosities). Although we found no difference in the density of vesicles in AIS and off-target varicosities at P28 (Fig. 5*G*; mean density of synaptic vesicles at P28^p^: AIS, 105 ± 5/µm^2^, *n* = 24 varicosities; off-target, 102 ± 13/µm^2^, *n* = 5 varicosities; Student’s *t* test: n.s. *p* = 0.78), the vesicle morphology was still significantly different between AIS and off-target varicosities (Fig. 5*I*; mean roundness at P28^q^: AIS, 0.758 ± 0.003, *n* = 24 varicosities, 661 vesicles; off-target, 0.81 ± 0.02, *n* = 5 varicosities, 116 vesicles; Student’s *t* test: ***p < 0.001). Taken together, these results suggest that synapse specificity of ChCs persists from early postnatal development to at least young adulthood.

## Discussion

Structured branching in target areas and subcellular synapse specificity are the most striking anatomic features of IN axons (Somogyi et al., 1998; Karube et al., 2004; Huang et al., 2007; Jiang et al., 2015). However, it has been unclear whether axonal organization and synaptic specificity of cortical INs are predetermined or established after refinement. Addressing these questions has been hampered by difficulties in tracking the development of homogenous IN subtypes with single-cell resolution. Here, by genetically targeting ChC progenitors, we established that developing ChCs form excessive branches and varicosities that eventually undergo pruning. Furthermore, we found that although ChCs form off-target varicosities as well as AIS varicosities, synapses are selectively formed at AIS varicosities during development and this synapse specificity persists into young adulthood. Our results provide evidence that ChCs establish subcellular synapse specificity in a predetermined manner while shaping axonal arbors through branch remodeling.

### Subcellular synapse specificity of ChCs is predetermined

Previous studies using classical dye-filling and genetic labeling revealed striking subcellular synapse specificity of IN subtypes in mature cortical circuits (Somogyi et al., 1998; Karube et al., 2004; Huang et al., 2007; Jiang et al., 2015). However, it has been an outstanding question how such an elaborate synaptic connectivity emerges during development. Ectopic formation and subsequent elimination of synapses are common strategies for neural circuits to refine connectivity based on the environment and corresponding computational requirements (Katz and Shatz, 1996; Sanes and Lichtman, 1999; Goda and Davis, 2003; Clarke and Barres, 2013; Hashimoto and Kano, 2013). Our finding that ChCs develop an excessive number of off-target varicosities, which undergo massive pruning, is seemingly consistent with this view because most axonal varicosities have been traditionally considered presynaptic structures (Meyer and Smith, 2006; Ruthazer et al., 2006; Wu et al., 2012). However, our analyses using immunohistochemistry, IEM, and exogenously introduced synaptic markers suggested that although forming varicosities both on and off AISs, ChCs predominantly develop presynapses at AIS varicosities throughout development and maintain this synaptic specificity in young adulthood. We cannot completely exclude the possibility that a small fraction of off-target varicosities form synapses as indeed, ∼10% of off-target varicosities contain presynaptic markers. However, it is hard to imagine that we missed early stage synapses ubiquitously formed at off-target varicosities because similar approaches were used in many other studies to identify nascent synapses (Zito et al., 2009; Wu et al., 2012). Thus, we conclude that subcellular synapse specificity of ChCs is predetermined. It is most likely that molecular cues localized at AISs control target recognition and/or synapse formation by ChCs. In the cerebellum, neurofascin (NF) is expressed in the soma and AIS of Purkinje cells and regulates navigation of basket cell (BC) axons from the soma to the AIS (Ango et al., 2004). Since NF is localized at the AISs of cortical PNs (Hedstrom et al., 2007), it would be of great interest to test whether NF also controls subcellular synapse specificity of ChCs.

### Potential roles of off-target varicosities in developing and young adult ChCs

Our finding of off-target, nonsynaptic varicosities in developing ChCs raises a question about their role in axonal wiring. Due to the following reasons, we predict that off-target varicosities may serve as initiation sites of filopodia, which are precursors of axonal branches. First, off-target varicosities and branch points concomitantly increase between P12 and P16. Second, axonal varicosities have been shown to serve as hotspots to generate filopodia in different systems including BC axons in the mouse cortex (Wu et al., 2012) and retinal axons in lower vertebrates (Meyer and Smith, 2006; Ruthazer et al., 2006). Third, we found that a significant fraction of off-target varicosities contain mitochondria, which have been shown to colocalize with branch points and play a role in branching in different types of neurons (Courchet et al., 2013; Spillane et al., 2013). Live imaging of developing ChCs will provide direct evidence for this hypothesis in the future.

Notably, our results showed that young adult ChCs (e.g., P28) retain off-target varicosities even after the pruning phase. Considering the completion of ChC axonal wiring at P21, we do not expect that these off-target varicosities actively form branches. This is not contradictory to our speculation discussed above if the competence of off-target varicosities to generate filopodia is developmentally downregulated. Recent studies showed that inhibitory INs in adult animals are highly plastic in response to changes in sensory experiences and natural learning and memory paradigms (Keck et al., 2011; van Versendaal et al., 2012; Trouche et al., 2013). Thus, an intriguing possibility is that off-target varicosities of young adult ChCs may serve as cellular devices for the formation of additional branches or synapses when activated by particular stimuli.

### ChC axonal arbors are established through remodeling

Developmental axonal refinement is widely observed in distinct nervous systems, including retinotectal axons, climbing fibers in the cerebellum, and motor axons at neuromuscular junctions (Katz and Shatz, 1996; Sanes and Lichtman, 1999; Goda and Davis, 2003; Clarke and Barres, 2013; Hashimoto and Kano, 2013). Axonal pruning usually occurs in parallel with synaptic pruning. Here we found that ChCs also undergo striking axonal remodeling to establish highly stereotyped axonal arbor organization with vertically aligned branches. However, interestingly, our data showed that the reduction in the number of branch points starts after ChCs largely complete formation of AIS synaptic varicosities. Based on this observation, we propose that axonal branches that failed to form synaptic varicosities may be preferentially pruned. Previous results showing that branch retractions halt at synaptic varicosities in developing axonal arbors (Meyer and Smith, 2006; Ruthazer et al., 2006) are consistent with this idea. Long-term live imaging of ChC axons and synaptic markers in a remodeling phase will provide a definitive answer to this issue.

Previous studies examined developmental processes of axonal arborization in BCs, which innervate perisomatic areas of PNs, and found that there is no obvious axonal overgrowth and pruning: they continuously increased branch numbers until reaching a plateau (Chattopadhyaya et al., 2004). However, because of cellular heterogeneity (Tremblay et al., 2016), substantial branch pruning in a single type of BCs could be hidden in the analysis. Thus, it remains elusive whether other morphologically distinct IN subtypes display developmental axonal remodeling like ChCs. Nevertheless, it would be interesting to speculate a potential reason why BCs and ChCs use different developmental processes to organize their axonal arbors. One major difference between BCs and ChCs is in the density of their postsynaptic subcellular targets. As proximal dendrites and cell bodies, which are typical subcellular targets of BCs, are densely intermingled in the cortical neuropil, axonal branches of BCs may easily find synaptic targets and form synaptic varicosities. Indeed, most axonal varicosities of BCs contain presynaptic markers at a light microscopic level (Wu et al., 2012). If synaptic varicosities control branch stability, axonal arbors of BCs would not undergo pruning. In contrast, because AISs are relatively sparsely distributed, a significant fraction of ChC branches may not be able to form synapses on subcellular targets. These branches free of synaptic varicosities could be retracted.

## Conclusion

In summary, we have provided the first evidence that ChCs establish axonal organization through remodeling but develop subcellular synapse specificity in a predetermined manner. Future studies of the molecular and cellular mechanisms of ChC wiring will give us important insight into how inhibitory circuits containing diverse IN subtypes are assembled during development. Consistent with their powerful influence on PN spike generation, ChCs have been implicated in the pathology of brain disorders such as schizophrenia (Lewis, 2010) and epilepsy (DeFelipe, 1999). Our results showing the developmental trajectory of ChC wiring may serve as a reference point to identify pathologic processes in mouse models of these brain diseases.

## Synthesis

Reviewing Editor: Siegrid Löwel, University of Goettingen Decisions are customarily a result of the Reviewing Editor and the peer reviewers coming together and discussing their recommendations until a consensus is reached. When revisions are invited, a fact-based synthesis statement explaining their decision and outlining what is needed to prepare a revision will be listed below. The following reviewer(s) agreed to reveal their identity: Jochen Staiger, Christiaan Levelt.

The manuscript is the first detailed description of the development of Chandelier cell synapses, asking how the subcellular specificity of Chandelier cells (ChC) that are thought to exclusively synapse on axon initial segments (AIS) of pyramidal cells is established during development. To answer this question the authors use recently developed mouse genetic and viral tools to sparsely and selectively label ChCs in the frontal cortex. With different anatomical and quantitative techniques they found that ChC axons show an initial exuberance of axonal branches and varicosities, which is followed by substantial postnatal pruning. Interestingly, varicosities apposed to AISs form inhibitory synapses while those that are not adjacent to AIS do not. These latter varicosities are less likely to be labeled by the synaptic markers VGAT and synaptophysin, and their vesicles are shaped differently. However, their surprising finding is that there are about 40% off-target varicosities, i.e. a huge fraction of the axon that does not synaptically target any postsynaptic profile, and that it is these varicosities/boutons that get preferentially/selectively pruned. At the same time, it seems that all axonal varicosities that form synapses do so right from the start and onto the correct subcellular structure, i.e. the axon initial segment for principal cells.

Basically, the study is carried out carefully and provides novel insights in the development of neocortical chandelier cells. While the manuscript is well written; both reviewers suggest that authors get help with linguistic editing.

The reviewers also agree that the interpretation that the non-synaptic varicosities may represent early stage branch points is somewhat surprising, considering their abundance at an age at which very little branching occurs. The reviewers ask the authors to expand their discussion considering the following arguments/ideas.

In Fig. 3E, it is shown that on P 28, i.e. close to adulthood and after pruning and remodeling, there are still around 40% off-target varicosities, which, if the single example in Fig. 5B is true and representative, would mean that 40% of all varicosities are non-functional! While it is possible that such varicosities could be the origins of branch points to remodel the axon, it is hard to imagine that ChC remodel 40% of their axon at any given time in adulthood. Please illustrate in a revised manuscript better resolved and uncolored original EM data to really show that these large varicosities do not form any synapses. Furthermore, it would be somewhat surprising that their numbers do not decrease after p21 when most axonal growth is expected to end. What about the possibility that these varicosities are in fact very early stage synaptic boutons that fail to mature/stabilize because the appropriate postsynaptic target is missing? Considering that inhibitory boutons (formed by other interneuron subsets) continue to be formed and lost into adulthood seem to make this option also quite likely.

Other issues:

The acronym SSC should be explained where it appears first (l. 164).

Replace "circularity" with "roundness".

Some expressions are hard to understand:

L. 276/277: every animal has only one brain, what does this sentence mean? Please rephrase.

Even more importantly, l. 305/306: do you mean horizontal axonal collaterals, or, primary axons - suggesting that multiple axons originate from one ChC? This is important because Fig. 2A indeed suggest that there are 2 separate axons. We are not sure how this potential confounder might have influenced the analysis of the study, i.e. have some dendritic branches (and their varicosities) been included in the study? Please clarify this issue and include it in the discussion.

Line 494: This sentence is hard to understand: "Second, axonal varicosities have been implicated in hotspots for filopodia initiation in different systems including basket cell axons in the mouse cortex (Wu et al., 2012) and retinal axons in lower vertebrates (Meyer and Smith, 2006; Ruthazer et al., 2006) although they are synaptic." What is meant here? Please rephrase and seek linguistic advice

L. 210: what are "... axonal structures that were co-localized with AnkG."?

The authors do not clearly explain why they perform transplantation experiments: to be able to transfect the cells with Syp-YFP? Please clarify/explicitly state the reason.

Image quality in Fig. 1C-G is not good enough (at least in PDF).

Fig. 2 (C) shows an enormous number of branch points in such a small sub-volume. How were they defined?

Line 230: "Xylasine" is misspelled (should be Xylazine).
