# Supplementary figures and images for: Neocortical Chandelier Cells Developmentally Shape Axonal Arbors through Reorganization but Establish Subcellular Synapse Specificity without Refinement
Source: eNeuro. 2017 May 12;4(3):ENEURO.0057-17.2017. doi: 10.1523/ENEURO.0057-17.2017 (PMC5458751; doi:10.1523/ENEURO.0057-17.2017)

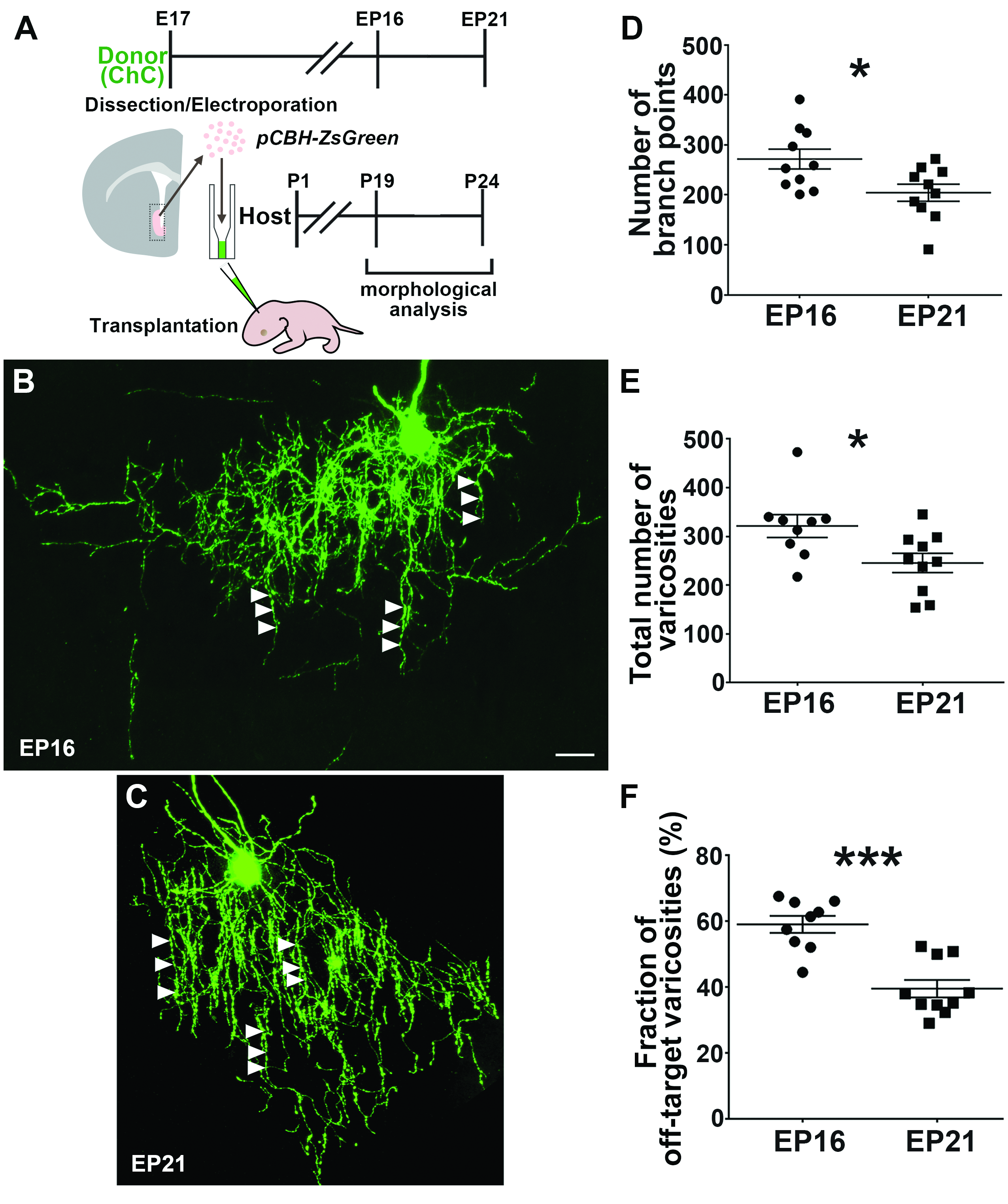

Supplement: Figure 4-1 [file enu003172309so8.tif]
